# Polymorphism-Aware Models in RevBayes: Species Trees, Disentangling Balancing Selection, and GC-Biased Gene Conversion

**DOI:** 10.1093/molbev/msae138

**Published:** 2024-07-09

**Authors:** Svitlana Braichenko, Rui Borges, Carolin Kosiol

**Affiliations:** Centre for Biological Diversity, School of Biology, University of St Andrews, Fife KY16 9TH, UK; Institute of Genetics and Cancer, University of Edinburgh, Edinburgh EH4 2XU, UK; Institut für Populationsgenetik, Vetmeduni Vienna, Wien 1210, Austria; Centre for Biological Diversity, School of Biology, University of St Andrews, Fife KY16 9TH, UK

**Keywords:** polymorphism-aware phylogenetic models, balancing selection, GC-biased gene conversion, Bayesian inference with MCMC, site frequency spectrum, species trees

## Abstract

The role of balancing selection is a long-standing evolutionary puzzle. Balancing selection is a crucial evolutionary process that maintains genetic variation (polymorphism) over extended periods of time; however, detecting it poses a significant challenge. Building upon the Polymorphism-aware phylogenetic Models (PoMos) framework rooted in the Moran model, we introduce a PoMoBalance model. This novel approach is designed to disentangle the interplay of mutation, genetic drift, and directional selection (GC-biased gene conversion), along with the previously unexplored balancing selection pressures on ultra-long timescales comparable with species divergence times by analyzing multi-individual genomic and phylogenetic divergence data. Implemented in the open-source RevBayes Bayesian framework, PoMoBalance offers a versatile tool for inferring phylogenetic trees as well as quantifying various selective pressures. The novel aspect of our approach in studying balancing selection lies in polymorphism-aware phylogenetic models’ ability to account for ancestral polymorphisms and incorporate parameters that measure frequency-dependent selection, allowing us to determine the strength of the effect and exact frequencies under selection. We implemented validation tests and assessed the model on the data simulated with SLiM and a custom Moran model simulator. Real sequence analysis of *Drosophila* populations reveals insights into the evolutionary dynamics of regions subject to frequency-dependent balancing selection, particularly in the context of sex-limited color dimorphism in *Drosophila erecta*.

## Introduction

Balancing selection (BS) represents a form of natural selection that maintains beneficial genetic diversity within populations (Bitarello et al. 2023). Multiple mechanisms contribute to maintaining variation, such as the heterozygote advantage or overdominance (heterozygous individuals having higher fitness), frequency-dependent selection (an individual’s fitness depends on the frequencies of other phenotypes or genotypes), antagonistic selection (in contexts like sexual conflicts or tissue-specific antagonism), and selection that changes through time or space in population. The evidence for BS is extensive, including examples from immune response such as the major histocompatibility complex (MHC) (Andrés et al. 2009; Spurgin and Richardson 2010; Bitarello et al. 2018), pathogen resistance (Bakker et al. 2006), plant and fungi self-incompatibility (Lawrence 2000; Castric and Vekemans 2004), and sex-related genes (Charlesworth 2004; Connallon and Clark 2014; Mank 2017; Kim et al. 2019).

BS finds its roots in the “balance hypothesis”, according to which populations exhibited high levels of diversity, with natural selection maintaining a balance among different alleles (Dobzhansky 1955). Historically, the classical theory diminished the ubiquity of the balancing hypothesis by explaining the evolution of populations through the interplay of mutations and purifying or positive selections with varying strengths. However, BS remains a valuable concept for explaining the persistence of polymorphisms over extended periods. According to Bitarello et al. (2023), three types of BS can be defined based on the acting timescales. Assuming the effective population size ($N_{e}=10^{6}$; Sprengelmeyer et al. 2020), generation time (10 days; Fernández-Moreno et al. 2007), and the divergence times between *Drosophila erecta* and *Drosophila orena* species ($3\times 10^{6}$ years; Yassin et al. 2016), which are studied here, one can translate these timescales into calendar times. In this context, BS can be categorized as ultra-long-term ($>3.7\times 10^{6}$ years), long-term ($>10^{5}$ years), and recent ($<10^{5}$ years).

The heterozygote advantage stands out as one of the initially proposed mechanisms for BS. The textbook example of this kind of BS is found in human African populations: homozygous individuals for the abnormal version of *β*-globin gene that makes hemoglobin are susceptible to sickle-cell disease, while heterozygous individuals exhibit resistance to malaria (Laval et al. 2019). In this study, even though we are capable of detecting heterozygote advantage as well, we focus more on another well-known mechanism of BS called negative frequency-dependent BS as defined by Charlesworth and Charlesworth (2010). This mechanism is observed when the fitness of one individual depends on the frequencies of other phenotypes or genotypes in the population. Very often, negative frequency-dependent selection manifests in the maintenance of one or several rare advantageous genotypes in a population. In the context of this study, we focus on ultra long-term BS (∼5 million years), which leads to sexual dimorphism in female *Drosophila erecta* resulting in the maintenance of dark and light females in the populations. The dark females are presumably engaging in mimicry among the males to avoid the costs associated with repeated matings (Yassin et al. 2016).

The role of BS has been a subject of considerable debate over the last century (Bitarello et al. 2023). With the advent of new sequencing technologies, there has been a renewed interest in this phenomenon. Some models, such as those based on heterozygote advantage and sexual antagonism, have been proposed by Connallon and Clark (2014) and Zeng et al. (2021). While these models are valuable for describing allele frequency dynamics in populations, they become impractical for inference due to the consideration of specific cases of BS that are challenging to generalize and increasing computational costs associated with expanding parameter space.

Thus, a model that is flexible enough to capture the intricate effects of BS yet simple is required for inferring frequency-dependent selection. Here, we propose a new model that incorporates BS and further integrates it into an inference approach. We build upon PoMos, a set of models developed over a decade for species tree inference (De Maio et al. 2013, 2015; Schrempf et al. 2016). A fast implementation of the PoMo approach for species tree inference is available in IQ-TREE (Schrempf et al. 2019).

Recently, PoMos were extended to account for directional selection (DS) and tested on the GC-biased gene conversion (gBGC; Borges et al. 2019; Borges and Kosiol 2020; Borges et al. 2022b, 2022a). This phenomenon is modeled similarly to DS, by setting relative fitnesses for *C* and *G* alleles higher than those for *A* and *T* alleles. Furthermore, in the inference setup, DS and gBGC are considered to be equivalent.

Borges et al. (2022b) demonstrated that including the effect of gBGC improves the accuracy of branch length estimation employed for molecular dating. Here, in the context of BS, we integrate the modeling of gBGC as it serves as the background force. This approach provides a more realistic null model, thereby enhancing the estimation of BS on experimental data.

PoMos prove to be valuable for modeling and detecting BS, as they are rooted in polymorphisms characterized by the prolonged existence of multiple genetic variations—markers of BS (Bitarello et al. 2023). This phenomenon manifests in a shift in the site frequency spectrum (SFS) toward an excess of intermediate frequency variants. These are sometimes identifiable by a peak in the intermediate frequencies of the SFS that cannot be explained by the interplay between mutation, genetic drift, and DS, as mentioned in Charlesworth (2006) and Charlesworth and Charlesworth (2010), but by BS. Consequently, these signatures are utilized by various frameworks to detect BS.

BS poses a significant challenge to detection methods due to its subtle nature, often entangled with structural variants and linkage disequilibrium (Charlesworth 2006; Fijarczyk and Babik 2015). Recent efforts have been made to propose universal and robust frameworks for BS detection. The software packages aimed at detecting BS are summarized in Table 1. These include methods based on genome scans with multiple summary statistics and composite likelihood ratio tests (CLRTs; Andrés et al. 2009; DeGiorgio et al. 2014; Bitarello et al. 2018; Cheng and DeGiorgio 2019, 2020, 2022), as well as deep-learning methods (Sheehan and Song 2016; Isildak et al. 2021; Korfmann et al. 2023). In Table 1, we summarize approaches that are most relevant to our study; for more details, please refer to Bitarello et al. (2023).

**Table 1.** Comparison of PoMos with other methods for detection of DS and BS

Function Package	Citation	Method	Multi-species	Trained and tested on human	DS	Detects magnitude and frequencies
BetaScan	Siewert and Voight (2017)	Summary statistics	−	+	−	−
	Siewert and Voight (2020)					
**BAL**ancing Selection	DeGiorgio et al. (2014)	CLRT	−	+	−	−
**L**ik**E**lihood **T**est						
NCD	Bitarello et al. (2018)	Summary statistics	−	−	−	−
MuteBaSS	Cheng and DeGiorgio (2019)	Summary statistics	+	−	−	−
MULLET	Cheng and DeGiorgio (2019)	CLRT	+	+	−	−
BalLeRMix	Cheng and DeGiorgio (2020)	CLRT	−	+	−	−
BalLeRMix+	Cheng and DeGiorgio (2022)	CLRT	−	+	+	−
**Ba**lancing **Se**lection	Isildak et al. (2021)	Deep learning	−	+	+	−
PoMos	Borges et al. (2019)	Bayesian inference	+	−	+	+
with selection	Borges et al. (2022a)					
(PoMoSelect + PoMoBalance)	This study					

The majority of the approaches mentioned above exploit long-term BS and are therefore focused on scenarios involving single species. Two exceptions to this are **MU**l**T**i-sp**E**cies **BA**lancing **S**election **S**ummaries (MuteBaSS) and **MUL**ti-species **L**ik**E**lihood **T**ests (MULLET) (Cheng and DeGiorgio 2019), which operate within the paradigm of ultra-long BS and accept multispecies data. Consequently, we utilize these packages for comparisons with our approach. Another aspect of the methods summarized in Table 1 is that the majority of them are trained and tested on human or great ape data. Therefore, one must exercise caution when applying them to other species. Moreover, unlike other approaches, Cheng and DeGiorgio (2022) strive to disentangle DS from BS. However, their approach requires intricate information about populations, such as recombination maps and ancestral pairwise alignment files.

By leveraging the advantages of accommodating multispecies data, applicability to most species (excluding bacteria and viruses), and incorporating mechanisms for disentangling DS from BS, our approach serves as a Bayesian inference tool. Our method not only detects selection but also quantifies its strength and frequencies, unlike most of the BS detection tools that show maximal performance at frequency equilibrium close to 0.5. Notably, **N**on-**C**entral **D**eviation (NCD; Bitarello et al. 2018) and subsequently MuteBaSS (Cheng and DeGiorgio 2019), which utilizes modified NCD statistics, possess a mechanism to detect BS at frequency equilibrium below 0.5. However, these frequencies must be pre-defined by the user. BetaScan2 (Siewert and Voight 2020) is also capable of detecting equilibrium frequencies, but when substitutions are specified, it is outperformed by NCD (Cheng and DeGiorgio 2019).

Evaluating the effect of BS remains challenging, requiring more model-based approaches (Fijarczyk and Babik 2015; Bitarello et al. 2023). Specifically, we require models that extend beyond heterozygote advantage, incorporating frequency-dependent selection and integrating both balancing and DS. Our method addresses these challenges in a particular manner. Currently, it focuses on single genes or groups of genes; however, it holds a high potential for parallel implementation. At the moment, it allows analyses across numerous individuals and populations over genomic regions, including several hundred base pairs. In the future, it is poised to enable genome-wide inferences.

## Materials and Methods

### Modeling the BS with PoMoBalance

**Fig. 1.**
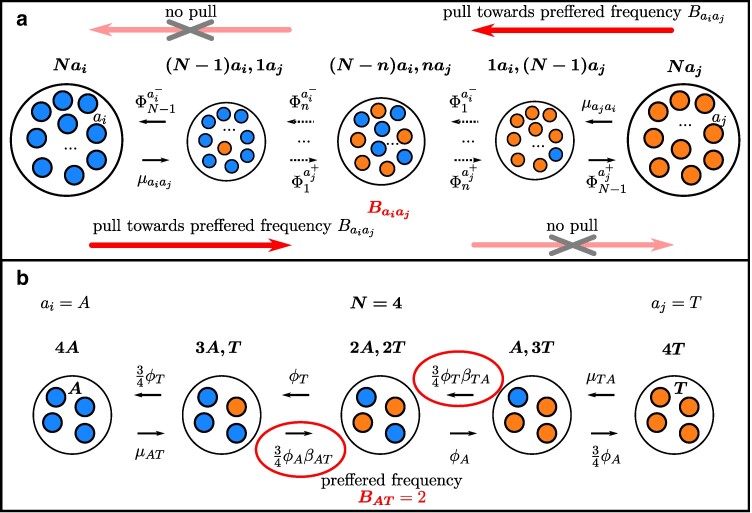
a) PoMoBalance model, presented as a Markov chain Moran-based model. The boundary states (monomorphic) are denoted by larger circles. These states encompass *N* individuals, with the left side showcasing individuals carrying the $a_{i}$ allele, and the right side representing individuals with the $a_{j}$ allele. In contrast, all the intermediate states, reflecting polymorphic conditions, are displayed using smaller circles. The transition rates from the monomorphic states are determined by mutation rates, whereas the transition rates from the polymorphic states are governed by the multiplicative fitness as indicated in Equation (1). Additionally, the multiplicative fitness encapsulates not only the DS effect but also the influence of BS, which exerts a force toward the state with the preferred allele frequency, $B_{a_{i}a_{j}}$, represented by dark arrows. If the transition occurs against this preferred state, there is no such attracting force, signified by the light crossed arrows. b) A specific instance of the PoMoBalance model, featuring a population size of N=4.

In this paper, we introduce the PoMoBalance model (depicted in Fig. 1a) that can be regarded as an extension of the PoMos with DS introduced by Borges et al. (2019), Borges and Kosiol (2020), Borges et al. (2022b), and Borges et al. (2022a). We will refer to the latter as PoMoSelect henceforth for brevity. Both PoMoSelect and PoMoBalance are distinguished in Table 2 and belong to the family of models known as PoMos that are continuous-time Markov chain models based on the Moran model (Moran 1958). The Moran model is a stochastic process that simulates a virtual population of *N* haploid individuals, with the power to incorporate boundary mutations and DS. Together with the Wright–Fisher model, they are both boundary mutation models. These models treat mutations as perturbations from the equilibrium state of populations, while selection drives population genotypes to fixation. The frequency-dependent formulation of such models makes them attractive for inference, since it is relatively easy to implement DS and BS in them. Moran model bears similarities to the Wright–Fisher model, which counts time in the number of generations. In contrast, the Moran model is continuous-time, measuring time in the number of births (Lanchier 2017). This characteristic makes the Moran model advantageous for phylogeny and experimental evolution approaches (Barata et al. 2023) that rely on a continuous-time paradigm.

**Table 2.** PoMo functions and parameters in RevBayes

Function (reference in the text)	Description	Parameters
fnPomoKN (nonreversible PoMoSelect or PoMoSelect)	Describes the evolution of a population with *K* alleles and *N* individuals subjected to mutational bias and selection	*K*, *N*, *μ*, *ϕ*
fnReversiblePomoKN (reversible PoMoSelect)	Particular case of PoMoKN when mutations are considered reversible	*K*, *N*, *π*, *ρ*, *ϕ*
fnPoMoBalanceKN (nonreversible PoMoBalance or PoMoBalance)	Describes the evolution of a population with *K* alleles and *N* individuals subjected to mutational bias, selection, and BS	*K*, *N*, *μ*, *ϕ*, *β*, *B*
fnReversiblePomoBalanceKN (reversible PoMoBalance)	Particular case of PoMoBalanceKN when mutations are considered reversible and the preferred frequency is in the middle B=N/2	*K*, *N*, *π*, *ρ*, *ϕ*, *β*

In this paper, we extend the Moran model to include BS in a four-allelic system representing the four nucleotide bases. The model encompasses 4+6(N−1) distinct states, with four monomorphic boundary states, denoting scenarios in which all individuals share the same allele. In contrast, the intermediate 6(N−1) states represent polymorphisms, where some individuals possess different alleles. Here, as shown in Fig. 1a, we consider only biallelic polymorphisms, where each state represents certain frequency *n* of alleles $a_{i}$ (monomorphic on the left) and N−n of $a_{j}$ (monomorphic on the right). These alleles signify four nucleotides i,j={A,C,G,T}. The combinations of alleles, indicated as $a_{i}a_{j}$, represent the possible pairs without repetition, namely *AC*, *AG*, *AT*, *CG*, *CT*, or *GT*.

The model incorporates mutation rates, $\mu_{a_{i}a_{j}}$ and $\mu_{a_{j}a_{i}}$ (as illustrated in Fig. 1a), which govern transitions from the monomorphic states, representing boundary mutations. The parameters of PoMos are defined in Table 3. Very often, the reversibility of the model is defined from certain symmetries in the parameters. In PoMoSelect, the mutation rates are presented as $\mu_{a_{i}a_{j}}=\rho_{a_{i}a_{j}}\pi_{a_{j}}$ and $\mu_{a_{j}a_{i}}=\rho_{a_{j}a_{i}}\pi_{a_{i}}$, similar to Tavare (1986). Parameters $\rho_{a_{j}a_{i}}$ are exhangeabilities of nucleotides (Yang 2014) that specify the relative rates of change between states *i* and *j*, and $\pi_{a_{j}}$ are nucleotide base frequencies, giving the equilibrium frequency at which each base occurs at all sites. If $\rho_{a_{i}a_{j}}=\rho_{a_{j}a_{i}}$, the model is reversible, otherwise, it is nonreversible.

**Table 3.** Parameters of PoMos in the four-allelic case

Parameter	Variable or vector	Description
$a_{i,j}$	*A*, *C*, *G*, *T*	Nucleotide bases
$a_{i}a_{\;j}$	*AC*, *AG*, *AT*, *CG*, *CT*, *GT*	Pairwise combinations of nucleotide bases
*N*	*N*	Effective population size
$\mu_{a_{i}a_{j}}$	$\mu =(\mu_{AC},\mu_{AG},\mu_{AT},\mu_{CG},\mu_{CT},\mu_{GT}$ ,	Mutation rates
	$\mu_{CA},\mu_{GA},\mu_{TA},\mu_{GC},\mu_{TC},\mu_{TG}$ )	
$\mu_{a_{i}a_{j}}=\rho_{a_{i}a_{j}}\pi_{a_{j}}$	$\mu =(\mu_{AC},\mu_{AG},\mu_{AT},\mu_{CG},\mu_{CT},\mu_{GT}$ )	Reversible mutation rates
$\pi_{a_{i,j}}$	$\pi =(\pi_{A},\pi_{C},\pi_{G},\pi_{T}$ )	Nucleotide base frequencies
$\rho_{a_{i}a_{j}}$	$\rho =(\rho_{AC},\rho_{AG},\rho_{AT},\rho_{CG},\rho_{CT},\rho_{GT}$ )	Exhangeabilities
$\phi_{a_{i,j}}$	$\phi =(\phi_{A},\phi_{C},\phi_{G},\phi_{T}$ )	Fitnesses
$\sigma_{a_{i,j}}=\phi_{a_{i,j}}-1$	$\sigma_{sel}=(\sigma_{A},\sigma_{C},\sigma_{G},\sigma_{T}$ )	Selection coefficients
	[if $\sigma_{A}=\sigma_{T}=0$, $\sigma =\sigma_{C}=\sigma_{G}$]	(GC-bias rate)
$\beta_{a_{i}a_{j}}$	$\beta =(\beta_{AC},\beta_{AG},\beta_{AT},\beta_{CG},\beta_{CT},\beta_{GT}$ )	Strength of BS
$B_{a_{i}a_{j}}$ [$B_{a_{i}a_{j}}/N$]	$B=(B_{AC},B_{AG},B_{AT},B_{CG},B_{CT},B_{GT}$ )	Preferred (equilibrium) frequencies of BS

In PoMoSelect, frequency shifts between polymorphic states are governed by genetic drift and DS favoring or disfavoring the reproduction of the $a_{i}$ allele. The fitness values are represented by $\phi_{a_{i}}=1+\sigma_{a_{i}}$, where $\sigma_{a_{i}}$ is a selection coefficient. In PoMoBalance, these frequency shifts additionally include BS transition rates that are regulated by a quantity that we call multiplicative fitness, expressed by the following equation for the selected state *n* as per Fig. 1a:


(1) $$\begin{array}{rl} \Phi_{n}^{a_{i,j}^{\mp }} & =\underset{\mathrm{drift}}{\underbrace{\frac{n(N-n)}{N}}}\overset{\mathrm{DS}}{\overbrace{\left(1+\sigma_{a_{i,j}}\right)}}\underset{\mathrm{BS}}{\underbrace{\beta_{a_{i}a_{j}}^{1/2\left[|n-B_{a_{i}a_{j}}|-|n\mp 1-B_{a_{i}a_{j}}|+1\right]}}},\\ & \;n=1,\ldots ,N-1, \end{array}$$

where there are three components: the first fraction corresponds to genetic drift or neutral mutations, the second multiplier represents DS, modeled similarly to previous PoMos. The final term in the form of a power-law function characterizes BS. This form of the BS term was derived phenomenologically from observations of various SFSs obtained from experimental data. It is governed by two key factors: the strength of BS, denoted as $\beta_{a_{i}a_{i}}$ (with $\beta_{a_{i}a_{i}}>0$), and a preferred frequency denoted as $B_{a_{i}a_{j}}$. The preferred frequency, a natural number within the range $0<B_{a_{i}a_{j}}<N$, designates the position of the polymorphic peak associated with BS in the SFS. Note that if $\beta_{a_{i}a_{i}}=1$, the resulting model aligns with the PoMoSelect model. We modeled BS in a frequency-dependent manner, in which the strength of BS governing the frequency shifts toward a favored frequency. The frequency equilibrium, as defined in Charlesworth and Charlesworth (2010), Bitarello et al. (2018), Bitarello et al. (2023), and Andrés et al. (2009), can be determined from our model as $B_{a_{i}a_{j}}/N$.

Reversibility criteria for PoMoBalance are different from those for the PoMoSelect model due to the higher complexity of the transition rates from the polymorphic states brought by BS terms. PoMoBalance is reversible only if exhangeabilities are symmetric and the preferred frequency is in the middle of the chain $B_{a_{i}a_{j}}=N/2$, where *N* is even (for more details, see supplementary material 1, Supplementary Material online).

Furthermore, we always assume that both $B_{a_{i}a_{j}}$ and $\beta_{a_{i}a_{i}}$ are symmetric. The strength of BS operates similarly to DS, but rather than favoring the fixation of alleles, it promotes the persistence of polymorphisms. In Fig. 1a, we visualize this additional attraction toward the preferred polymorphic state with dark arrows when $\beta_{a_{i}a_{i}}>1$. After replacing variables and simplifying the expressions with power terms, the transition rates become $\Phi_{n}^{a_{i,j}^{\mp }}=(n(N-n)/N)\phi_{a_{i,j}}\beta_{a_{i}a_{j}}$, if $n<B_{a_{i}a_{j}}$, and the absence of the BS attractor is indicated with light crossed arrows in the figure when $\Phi_{n}^{a_{i,j}^{\mp }}=(n(N-n)/N)\phi_{a_{i,j}}$, if $n\geq B_{a_{i}a_{j}}$. To provide a more concrete example, we represent the transition rates of a population with N=4 individuals in Fig. 1b, where the preferred frequency is B=2. It is important to note that in cases where $\beta_{a_{i}a_{i}}<1$, we do not model BS, but instead a form of purging selection occurs that leads to the removal of polymorphisms more than expected by drift (for a detailed explanation, see supplementary material 1, Supplementary Material online).

In the broader context, the PoMoBalance model can be characterized through the instantaneous rate matrix denoted as *Q*, where each specific transition rate within the model corresponds to an element of this matrix


(2) $$\begin{array}{rl} & q^{\left\{na_{i},(N-n)a_{j}\}\to \{ma_{i},(N-m)a_{j}\right\}}=\left\{ \begin{array}{cc} \;\mu_{a_{i}a_{j}} & \;\text{if}\;n=N,m=N-1,\\ \;\mu_{a_{j}a_{i}} & \;\text{if}\;n=0,m=1,\\ \frac{n(N-n)}{N}(1+\sigma_{a_{i}})\beta_{a_{i}a_{j}}^{1/2\left[|n-B_{a_{i}a_{j}}|-|n+1-B_{a_{i}a_{j}}|+1\right]} & \text{if}\;m=n+1,\\ \frac{n(N-n)}{N}(1+\sigma_{a_{j}})\beta_{a_{i}a_{j}}^{1/2\left[|n-B_{a_{i}a_{j}}|-|n-1-B_{a_{i}a_{j}}|+1\right]} & \text{if}\;m=n-1,\\ \;0 & \;\text{if}\;|m-n|>1, \end{array}\right. \end{array}$$

where the variables *n* and *m* represent neighboring states as illustrated in Fig. 1a. This matrix summarizes the PoMoBalance model, depicting transitions from monomorphic states regulated by mutation rates and from polymorphic states governed by Equation (1). Since PoMoBalance is the Moran-based model, the allele frequency shifts exceeding one are prohibited, as specified in the final condition outlined in Equation (2). The diagonal elements of this matrix are determined such that the sum of each respective row is equal to zero.

Both the PoMoSelect and PoMoBalance models have been incorporated into a Bayesian phylogenetic inference framework RevBayes (Höhna et al. 2016, 2017, 2018; Borges et al. 2022a), available at https://revbayes.github.io/, employing a probabilistic graphical model representation.

### Bayesian Inference Using PoMoBalance with RevBayes

The advantage of using RevBayes for implementing PoMos is the flexibility of the use of probabilistic graphical models allowing us to combine complex models while taking advantage of communicating them with users through extensive tutorials and discussion forums. RevBayes employs a Bayesian inference based on the Markov chain Monte Carlo (MCMC) sampler and it is an open-source framework for phylogenetic inference, molecular dating, discrete morphology, and ancestral state reconstruction (Höhna et al. 2016, 2017, 2018; Borges et al. 2022a). Our integration of PoMoBalance into RevBayes enables users to perform phylogenetic tree inference, DS analysis, and now, identify BS. Unlike previous methods for detecting BS discussed earlier, our software not only detects BS but also quantifies its strength and identifies the alleles and their frequencies under selection. For detailed instructions on implementing RevBayes scripts with PoMoBalance, please refer to the PoMoBalance tutorial available at https://revbayes.github.io/tutorials/pomobalance/.

In PoMos’ data input, count files are employed, which can be generated from format for nucleotide ( FASTA) sequences of multiple individuals and species or VCF (Variant Call Format) files with the corresponding reference using the cflib package available on GitHub at https://github.com/pomo-dev/cflib (Schrempf et al. 2016). Additionally, we include scripts to correct for sampling biases, which can be helpful when the number of individuals sampled from populations varies and when it differs from the PoMo population size. These biases may emerge from undersampling genetic diversity, where polymorphic sites sampled from larger populations may erroneously appear monomorphic. To address this, the binomial sampling method, as initially proposed by Schrempf et al. (2016), assists in smoothing out sampling biases at the tips of a phylogenetic tree.

Additionally, PoMoSelect includes a rescaling tool for adjusting inferred parameters across different population sizes. Parameters calculated in the PoMos, originally in terms of virtual population sizes, can be rescaled to represent the actual population sizes. This rescaling is achieved using the mapping method introduced by Borges et al. (2019) and explained in the context of PoMoBalance in supplementary material 2, Supplementary Material online.

RevBayes offers several PoMo functions tailored to different inference scenarios, including fnPomoKN, fnReversiblePomoKN, fnPomoBalanceKN, and fnReversiblePomoBalanceKN. The first two functions are discussed in detail by Borges et al. (2022a). The roles and input parameters for each function are summarized in Table 2.

They are designed to infer data from *K* alleles, with the most common scenario involving K=4, although other options (e.g. K=2) are also available. Additionally, RevBayes accommodates the parameters of the PoMoBalance model outlined in section “Modeling the BS with PoMoBalance” and Table 3. These include the virtual population size *N*, mutation rates *μ* represented through nucleotide base frequencies *π*, and exhangeabilities *ρ* in the reversible case. Additionally, it includes a vector encompassing allele fitnesses *ϕ*, which, in our case, reflects gBGC as previously studied by Borges et al. (2019). We sometimes mention DS and gBGC interchangeably since the latter is modeled similarly to DS, with higher relative fitness for *C* and *G* alleles compared to *A* and *T* alleles. It is represented by the vector ϕ=(1,1+σ,1+  σ,1), where *σ* is a GC-bias rate. We also define two vectors for the strength and location of the BS peak for each combination of alleles *β* and *B*.

**Fig. 2.**
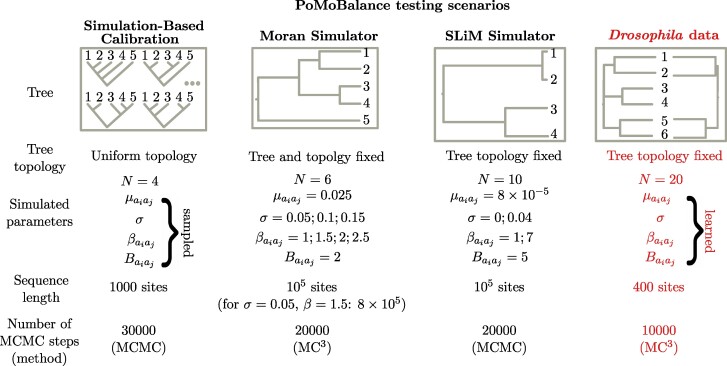
Testing scenarios for PoMoBalance include various types of trees, tree topologies, parameters of PoMos utilized in the tests, sequence lengths, and the number of MCMC steps. Simulation-based calibration involves data simulated under 1,000 parameters sampled from priors, while the Moran and SLiM frameworks also rely on simulated data for several values of *σ* and *β*. Additionally, we employ experimental data extracted from various subspecies of *Drosophila*.

For the Bayesian inferences conducted here, we employ dnDirichlet priors (concentration 0.25 for all alleles) on base frequencies *π* and mvBetaSimplex moves due to their sum-to-unity nature. For *ρ*, *σ*, and *β*, dnExponential priors are chosen as appropriate priors for positive real parameters with rates 10, 10, and 1, respectively, similar for all combinations of alleles. We use standard mvScale moves for these variables, but if they exhibit correlation, we may introduce additional moves like mvUpDownScale, mvAVMVN, mvSlice, or mvEllipticalSliceSamplingSimple to mitigate the correlation. In some cases, we observed a correlation between *σ* and *β*, and incorporating the mvAVMVN move helped to resolve it for some chains. The preferred frequency *B* is a positive natural number within the range 0<B<N, and Uniform priors in this range are set. The variable is rounded on each MCMC step to obtain discrete results. We introduce two moves, mvSlide and mvScale, to enhance parameter space exploration. Such a technique leads to faster convergence compared to UniformNatural prior and discrete variable moves. We assign different weights to each move; however, the specific values are less critical since autotuning of weights occurs during the MCMC burn-in period. Our analysis involves running both the Metropolis–Hastings MCMC sampler (mcmc), and where relevant, the Metropolis-coupled MCMC sampler or MC^3^ (mcmcmc), which includes high-temperature and cold chains to overcome local minima. Both versions normally run four parallel chains to ensure convergence. The number of MCMC steps required for convergence (ESS > 200) for different types of analyses is depicted in Fig. 2.

### Data Simulation, Analysis, and Inference

Extensive testing of PoMoBalance has been conducted across multiple scenarios, employing data simulated through different techniques. Each scenario is summarized in Fig. 2.

First, we conducted a built-in validation analysis within RevBayes. This analysis is based on the simulation-based calibration procedure (Talts et al. 2020), the approach used to test the accuracy of parameter inference through the following steps:

1. Drawing 1,000 random parameter values and a random five-species trees with uniform topology from the priors.
2. For each drawn parameter value simulating data sample with 1,000 nucleotide sites.
3. Performing MCMC inference for each sample.
4. Calculating coverage probabilities.

Coverage probabilities (Talts et al. 2020) are estimated based on the observation that 90% (or any arbitrary percentage) of credible intervals obtained with MCMC should contain the simulated parameter value in 90% of the samples. Simulation-based calibration leverages the frequentist properties of Bayesian inference. The advantage of this approach is its ability to simultaneously test the model across various parameters and multiple five-species trees. Additionally, we calculate the scores for tree topology, measured by mean Robinson–Foulds distances (Höhna et al. 2018), inferring tree topologies especially for large trees known to be a notoriously challenging task (Cavalli-Sforza and Edwards 1967). The deliberate choice of a small virtual population size, N=4, aims to test our models with minimal computational cost, as previous findings suggest that performance tends to remain consistent even with an increase in *N* (Borges et al., unpublished). We also conducted tests with N=6, yielding similar performance. However, testing with higher values of *N* becomes challenging due to the increasing computational cost associated with larger values. Nonetheless, we anticipate that the performance would remain consistent.

**Fig. 3.**
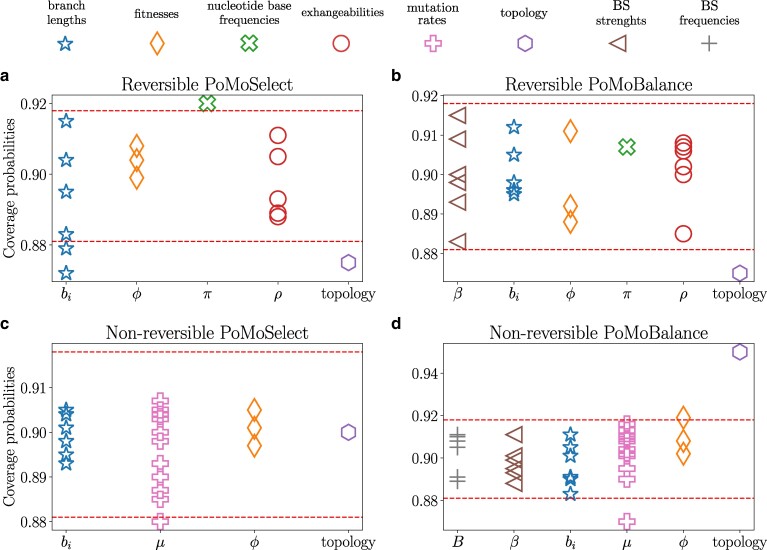
Coverage probabilities determined through validation analysis within RevBayes, employing distinct computational routines for reversible scenarios: a) PoMoSelect and b) PoMoBalance, as well as for nonreversible scenarios: c) PoMoSelect and d) PoMoBalance. The dashed lines indicate 90% CIs and fixed virtual population size for all cases was N=4.

**Fig. 4.**
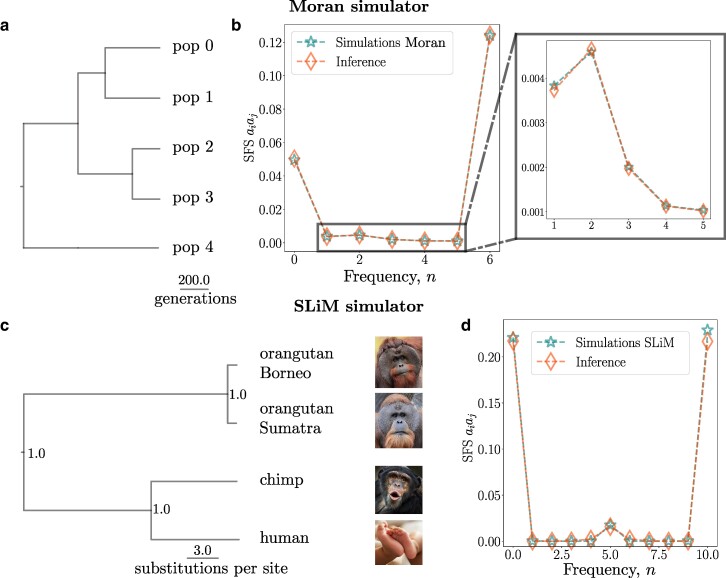
a) Phylogenetic tree simulated using the Moran simulator within RevBayes, the branch lengths are expressed in numbers of generations; the tree remains fixed for these analyses. b) SFS of the data with BS simulated using the Moran model with N=6 (stars), with the tree from (a) exhibiting good agreement with the SFS obtained from the inference using PoMoBalance (diamonds); the inset magnifies the BS peak. c) Phylogenetic tree of great apes simulated with SLiM and subsequently inferred with RevBayes, the branch lengths are expressed in the number of substitutions per site. Posterior probabilities are indicated at the nodes. Images are distributed under a Creative Commons license from Wikimedia and Microsoft. d) Comparison of the SFS with N=10, akin to (b), obtained from the simulated data with SLiM and the tree from (c). The SFS representation ($a_{i}a_{j}$) includes *AC*, *AG*, *AT*, *CG*, *CT*, and *GT*, demonstrating similarity in all cases.

Subsequently, a custom five-species tree (refer to Figs. 2 and 4a) was simulated using a Moran simulator in RevBayes. In this analysis, we utilize a five-species tree, as most methods for detecting ultra-long-term BS focus on testing fixed trees with four species or fewer (Cheng and DeGiorgio 2019). Our simulations cover timescales associated with long-term or ultra-long-term BS, as this example is not tied to any specific species. Here, we maintain the tree fixed to ensure better performance of the method. We recommend employing PoMoSelect for tree inference initially, as it has demonstrated better performance in inferring tree topologies (refer to Fig. 3). In testing the PoMoBalance approach, our focus is primarily on inferring gBGC and BS parameters. We simulate the sequences under the same model to ensure the precise recovery of parameters from data simulated under the similar models but in diverse evolutionary settings, including drift, gBGC, BS, and a combination of BS and gBGC. For most of the values, we simulated $10^{5}$ genomics sites, while for the intertwined scenario of weak BS ($\beta_{a_{i}a_{j}}=1.5$) and gBGC (σ=0.05), we required $8\times 10^{5}$ to achieve satisfactory convergence.

Furthermore, we assessed the performance of our package using data simulated within the evolutionary framework SLiM (Haller and Messer 2019). In this test, we used a tree including four great ape species: orangutans from Borneo and Sumatra islands, chimpanzees, and humans (refer to Figs. 2 and 4c and supplementary Fig. S1, Supplementary Material online). This tree had been previously estimated without BS using PoMos by Schrempf et al. (2016). In this setup, we simulate ultra-long-term BS and we first infer the tree with PoMoSelect. Subsequently, for the PoMoBalance analysis, we maintain the tree topology fixed and infer tree branch lengths alongside other parameters. The great ape species are of particular interest in the context of our paper as they exhibit several well-documented instances of BS, such as those observed in the MHC locus (Cagan et al. 2016). Another classical example of heterozygote advantage is sickle-cell disease, extensively studied in humans, however, its role in other great ape species remains a subject of debate (Laval et al. 2019). In SLiM simulations, we implemented heterozygote advantage within the great apes tree to simulate BS. Unlike the Moran simulator, SLiM simulations incorporated three regimes: drift, gBGC, and BS, excluding combination of BS and gBGC. This adjustment was necessary due to the heterozygote advantage overpowering gBGC in SLiM. Other features not explicitly considered by the Moran model but simulated in SLiM are genetic recombination and demography. Refer to supplementary material 3, Supplementary Material online, for more details on SLiM simulations.

Following this, we applied PoMoBalance to real datasets exhibiting BS associated with sexual dimorphism in *Drosophila erecta* females (Yassin et al. 2016). This case was chosen to exemplify ultra-long-term negative frequency-dependent BS in sexual selection, a topic of increasing interest (Croze et al. 2017). Please refer to Fig. 2, for details of the inference, and data availability details. Sequences were obtained for the *tan* gene in the $t_{\mathrm{MSE}}$ region. In addition to *Drosophila erecta* dark (seven individuals) and light (nine individuals), we extract data of multiple individuals from four closely related subspecies: *D. santomea* (10 individuals), *yakuba* (15 individuals), *melanogaster* (22 individuals), and *simulans* (18 individuals). We inferred trees in two cases: when all six subspecies were involved, and in the four-subspecies case, where we discarded *D. santomea* and *yakuba* due to poor quality of sequences. We performed the sequence alignment using MAFFT software (Rozewicki et al. 2019), filtered out sites containing more than 50% missing data and converted them into count files using the cflib package (Schrempf et al. 2016). The final sequences contained ∼400 sites. For the neutrality analyses performed with Tajima’s D (Tajima 1989) and Hudson-Kreitman-Aguade (HKA)- like (Begun et al. 2007) tests, we also used 5-kb upstream (∼400 sites) and 10-kb downstream (∼900 sites) regions that are known to be neutral. The data analysis pipeline is available in the supplementary repository (https://github.com/sb2g14/PoMoBalance).

## Results

### Validation Analysis for PoMoSelect and PoMoBalance

To validate the implementations of PoMoSelect and PoMoBalance, as depicted in Fig. 3, we employ the simulation-based calibration procedure implemented in RevBayes (Talts et al. 2020). In our study, we evaluate both the PoMoSelect model with DS proposed previously by Borges et al. (2022a) and the model that incorporates directional and BS (PoMoBalance), as outlined in section “Modelling the BS with PoMoBalance”.

In Fig. 3, we conduct simulation-based calibration for four PoMo functions (see Table 2) in both reversible and nonreversible implementations, simulating the trees with five taxa and a uniform topology. The markers in the figure represent coverage probabilities for various parameters, including tree branch lengths (stars), fitnesses (*ϕ*, diamonds), nucleotide base frequencies (*π*, X unfilled), exhangeabilities (*ρ*, circles), mutation rates (*μ*, pluses unfilled), BS strengths (*β*, triangles left), preferred frequencies (*B*, pluses), and topology (octagons). Different marker types distinguish values corresponding to different alleles or their combinations as per Table 3. Notably, nucleotide base frequencies exhibit a single coverage probability due to their origin from dnDirichlet. For fitnesses, they are relative by definition, with one of them always taking value of 1. Therefore, three coverage probabilities are observed instead of four. The 90% confidence bounds for MCMC are shown by red dashed lines. The scores for topologies and branch lengths are best estimated for the nonreversible PoMoSelect, presumably it has fewer degrees of freedom, reducing the likelihood of encountering local minima during inference. Therefore, in this paper, we adhere to a combined approach using PoMoSelect for tree or tree topology estimation and PoMoBalance for estimating gBGC and BS.

Despite using a small virtual population size (N=4) for computational efficiency, the majority of coverage probabilities lie within or very close to the confidence bounds, ensuring the validity of the implementations. The analysis of larger population sizes (N=6) has shown equivalent performance.

### Testing PoMoBalance on the Data Generated with Moran and SLiM Simulators

**Fig. 5.**
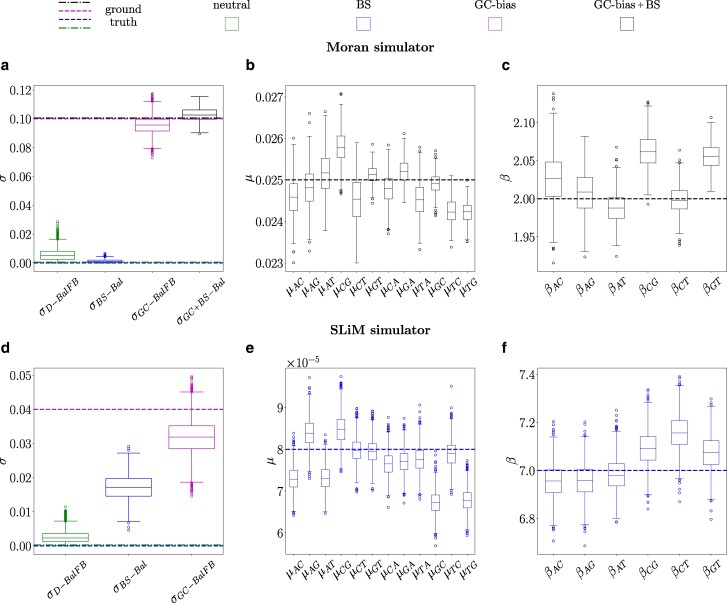
Posterior distributions of inferred parameters compared to their expected values. Subplots a), b), and c) employ the Moran model simulator, in Fig. 4a and b. Conversely, subplots d), e), and f) use the SLiM simulator, corresponding to Fig. 4c and d. Data simulations encompass four regimes: D for drift, GC for gBGC, BS for balancing selection, and GC + BS for the combination of gBGC and BS. Inference methods include BalFB, representing inference with PoMoBalance while fixing preferred frequencies *B*, and Bal, representing regular inference with PoMoBalance. True values are indicated by dashed and dot-dashed lines. a) Posterior plots for the GC-bias rate *σ*, with two boxplots on the left indicating simulated data in regime D inferred with BalFB and BS inferred with Bal. Two boxplots on the left show distributions that correspond to regime GC inferred with BalFB and GC + BS inferred with Bal. b) Estimates for mutation rates, and c) strengths of BS in the simulation scenario GC + BS. d) Posterior plots for SLiM data inference in three simulation regimes D, BS, and GC, analogous to (a), indicating the GC-bias rate *σ*. e) Estimates for mutation rates and f) strengths of BS corresponding to the BS simulation scenario in SLiM.

**Fig. 6.**
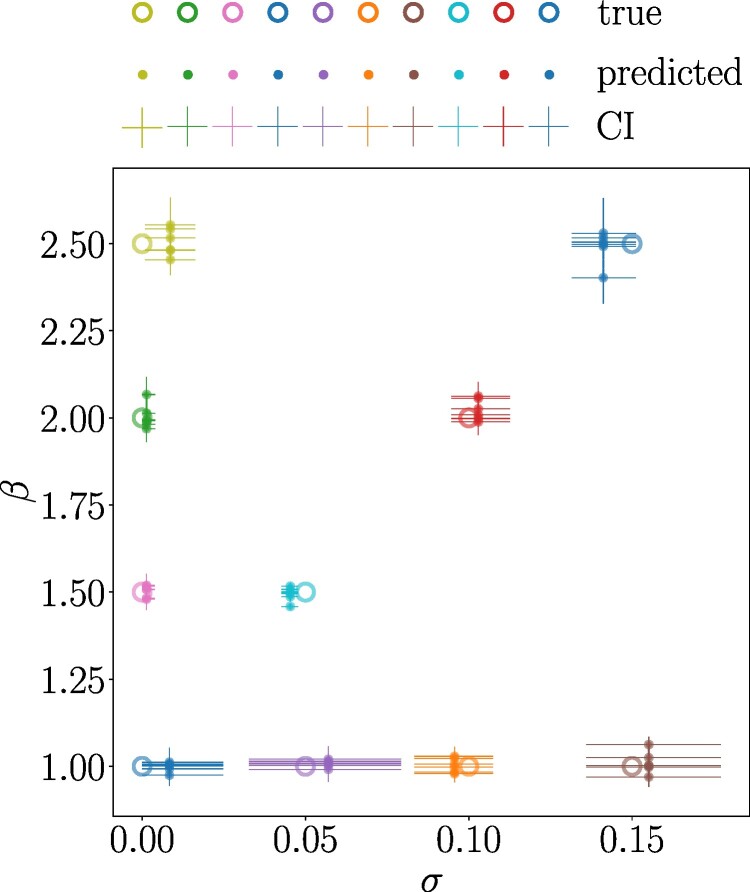
Testing PoMoBalance in a range of GC-bias rate *σ* and strength of BS *β* on the data generated with the Moran model. Large open markers represent true values, smaller closed markers with error bars correspond to the mean values of posterior predictions by PoMoBalance and their 95%CI, respectively.

In this subsection, we assess the performance of the PoMoBalance model using data simulated under various evolutionary scenarios with two different simulators. The details for the data generated with the first simulator, referred to as the Moran simulator, are depicted in Figs. 2, 4a,b, and 5a–c. In this analysis, we utilize RevBayes and our PoMoBalance implementation to simulate PoMo states from the nonreversible Moran model for generality, employing pre-selected parameter values akin to the scenario described in the previous subsection. However, in this case, we employ a custom phylogenetic tree depicted in Fig. 4a, use only a few parameter sets (shown in Fig. 2), and omit the calculation of coverage probabilities. Instead, we evaluate how far the inferred values deviate from the true values for a range of *σ* and *β*, as illustrated in Fig. 6. Note that the accuracy of the inference decreases and confidence intervals (CIs) increase with an increase in *σ* and *β*, but still the latter intersect the true values. In the case where σ=0.05 and β=1.5, we had to increase the number of sites from $10^{5}$ to $8\times 10^{5}$ for better convergence. The CIs are the largest for β=1, corresponding to the case where there is no BS, leading to significant uncertainty in learning preferred frequencies, which affects other parameters.

Additionally, we compare the SFS for σ=0.1 and β=2 in Fig. 4b, calculated from the simulated data depicted by stars, with theoretical predictions derived using parameters inferred with PoMoBalance illustrated with diamonds. The SFSs agree quite well despite slight inaccuracies in inferring parameters. The theoretical predictions are estimated numerically from the PoMo matrix in Equation (2), using the Markovian property dP(t)/  dt=P(t)Q, where P(t)=exp(tQ). By matrix exponentiation at very long times ($t=10^{6}$), we obtain the stationary distribution for the PoMo states, which coincides with the SFS. Further details about stationary frequencies in the PoMoBalance model can be found in supplementary Fig. S1, Supplementary Material online.

Figure 5a–c depicts boxes and whiskers of the posterior distributions derived from MCMC inference with the data simulated with the Moran model. The data are simulated under four evolutionary regimes: D for neutral mutations or drift, GC for GC-biased gene conversion (gBGC), BS for balancing selection, and GC + BS for the combination of gBGC and BS. We plot the boxes alongside the ground truth parameters (dashed for gBGC and BS, dotted-dashed for neutral and gBGC + BS) for comparison. Refer to supplementary Table S1, Supplementary Material online, for posterior means and CIs for selected points. Figure 4b illustrates the SFS for the last case. In the estimation of the posterior in all cases, we discard the MCMC burn-in period.

Within the box plots in Fig. 5a, we display estimates for the GC-bias rate in all four regimes, which align well with the true values. Mutation rates are shown in Fig. 5b, and BS strengths are depicted in Fig. 5c focusing solely on the GC+ BS regime for brevity. Posterior plots for preferred frequencies are not presented due to spike-like distributions as MCMC chains converge to the true values $B_{a_{i}a_{j}}=2$ during the burn-in period. This corresponds to the BS peak in Fig. 4b inset.

In Figs. 4c,d and 5d–f, we utilize the evolutionary simulation framework SLiM proposed by Haller and Messer (2019). For this simulation, we employed the great apes tree in supplementary Fig. S2, Supplementary Material online, implementing heterozygote advantage with SLiM (see supplementary material 3, Supplementary Material online, for details). The tree inferred with RevBayes in Fig. 4c is comparable to the simulated tree, with posterior probabilities at each node equal to 1. The SFS in Fig. 4c is extracted from the data and features a well-distinguished peak that is effectively captured by the inference.

In SLiM simulations, we implemented three regimes (D, GC, and BS). The posterior distributions for GC-bias rate in these regimes are illustrated in Fig. 5d. We obtain reasonable estimates in the D and GC regimes, but in the BS regime, *σ* is overestimated. This occurrence is due to the challenge of distinguishing *σ* and *π* for small virtual populations. While not easily discernible in the mutation rates presented in Fig. 5e, it becomes apparent when examining the inferred nucleotide base frequencies *π* (refer to supplementary Table S2, Supplementary Material online). Increasing the virtual PoMo size to N=20 resolves this problem partially resulting in much lower $\sigma_{\mathrm{BS}\text{-}\mathrm{Bal}}=0.008$. In this analysis, our focus is on the estimation of BS strength, which shows promising results in Fig. 5e. The preferred frequencies are also inferred accurately, similar to the Moran simulator.

Additionally, in Table 4, we present scaled scores obtained from tests conducted with MuteBaSS (${\text{HKA}}_{\mathrm{trans}}$, NCD, ${\text{NCD}}_{\mathrm{opt}}$, ${\text{NCD}}_{\mathrm{sub}}$) and MULLET (${\text{T}}_{1\text{trans}}$, ${\text{T}}_{2\text{trans}}$) (Cheng and DeGiorgio 2019). The scores for summary statistics and likelihood-based methods were calculated using the sliding windows approach, while our method is evaluated through the logarithm of the Bayes factor (BF).

**Table 4.** Scaled by the scores calculated in the neutral case tests run with MuteBaSS (${\text{HKA}}_{\mathrm{trans}}$, NCD, ${\text{NCD}}_{\mathrm{opt}}$, ${\text{NCD}}_{\mathrm{sub}}$) and MULLET (${\text{T}}_{1\text{trans}}$, ${\text{T}}_{2\text{trans}}$) (Cheng and DeGiorgio 2019), obtained by averaging the scores in sliding window analyses with optimal window sizes and a shift of 10 nucleotides vs log(BF) calculated from PoMoBalance inference

Scaled score SLiM data	$\left\|{\text{HKA}}_{\mathrm{trans}}\right\|$	‖NCD‖	$\left\|{\text{NCD}}_{\mathrm{opt}}\right\|$	$\left\|{\text{NCD}}_{\mathrm{sub}}\right\|$	$\left\|{\text{T}}_{1\text{trans}}\right\|$	$\left\|{\text{T}}_{2\text{trans}}\right\|$	‖log(BF)‖
Drift	1.0	1.0	1.0	1.0	1.0	1.0	1.0
GC-bias	0.07	1.0018	1.003	1.004	1.01	0.94	1.01
BS	12.68	1.0024	1.012	1.057	2.26	4.44	146.05

The data were generated with SLiM on the tree shown in Fig. 4c under neutral conditions, with gBGC or BS.

The data were generated via SLiM, similarly to Figs. 4c and 6d–f under drift, gBGC, and BS regimes. For the details of the calculations, please refer to supplementary material 4, Supplementary Material online.

The strongest evidence of BS is indicated by our method (log(BF)), followed by ${\text{HKA}}_{\mathrm{trans}}$ and ${\text{T}}_{2\text{trans}}$. However, the scores of ${\text{HKA}}_{\mathrm{trans}}$ are highly dependent on the window sizes. Please note that these results must be interpreted with caution, as the scores are calculated for different approaches operating on different scales.

### Detection of BS in ***Drosophila erecta***

In this analysis, we examine sequences derived from experimental genomic data of various *Drosophila* subspecies. We specifically explore the example of sexual dimorphism in the $t_{\mathrm{MSE}}$ gene region, featuring the *tan* gene observed in *Drosophila erecta* females, as studied by Yassin et al. (2016). Table 5 presents the results of Tajima’s D (Tajima 1989), HKA-like (Begun et al. 2007), and ${\text{HKA}}_{\mathrm{trans}}$ (Cheng and DeGiorgio 2019) tests indicating the potential presence of BS in the $t_{\mathrm{MSE}}$ region in contrast to neutral sequences 5-kb upstream and 10-kb downstream from the region.

**Table 5.** Results of Tajima’s D and HKA-like tests include the number of polymorphic sites (Pol) between dark and light *Drosophila erecta* lines and divergent (Div) sites between both *erecta* lines and *Drosophila orena* in the $t_{\mathrm{MSE}}$ region, along with two neutral regions

Gene region	Tajima’s D	Pol	Div	Pol/Div	${\text{HKA}}_{\mathrm{trans}}$
$t_{\mathrm{MSE}}$	3.99	51	28.5	1.78	0.031
5-kb upstream	− 1.1	40	51.9	0.77	$-6.5\times 10^{-5}$
10-kb downstream	0.88	32	33.5	0.95	− 0.175

The ${\text{HKA}}_{\mathrm{trans}}$ method is performed with MuteBaSS on *Drosophila erecta* (dark and light variants), *melanogaster*, and *simulans* by averaging scores within 700-nucleotide windows with a step size of 10 nucleotides.

The conclusion is drawn from a significant elevation of Tajimas D in the region of interest. Regarding the HKA-like test, we observe a notably higher proportion of polymorphic sites (Pol) between dark and light *Drosophila erecta* lines compared to divergent (Div) sites between both *erecta* lines and *Drosophila orena*, a closely related species to *erecta*. This increased polymorphism suggests the presence of BS. However, the $\chi^{2}$ test performed on these short sequences does not yield a significant result. In Yassin et al. (2016), the test is conducted on longer sequences containing the $t_{\mathrm{MSE}}$ region and leads to a significant result. The ${\text{HKA}}_{\mathrm{trans}}$ method is executed using MuteBaSS on *Drosophila erecta* (dark and light variants), *melanogaster*, and *simulans*. Negative scores for the upstream and downstream regions indicate the absence of BS, unlike the positive score for the $t_{\mathrm{MSE}}$ region, confirming the presence of BS.

**Fig. 7.**
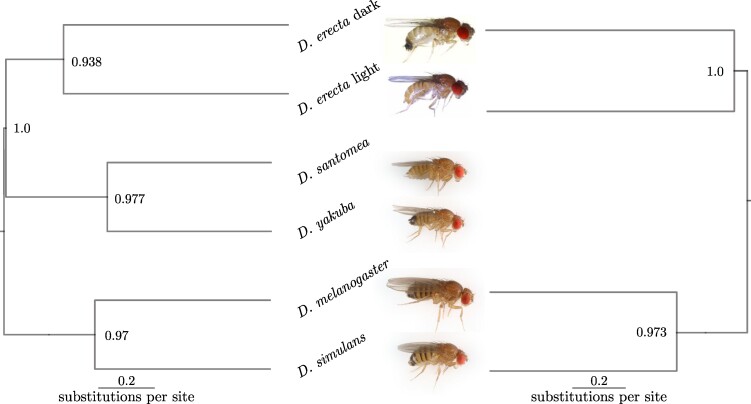
Phylogenetic tree inferred from the sequencing data obtained in the $t_{\mathrm{MSE}}$ region across six (left) and four (right) subspecies of *Drosophila*. Posterior probabilities are indicated at the nodes. Images of *D. santomea, yakuba, melanogaster*, and *simulans* are credited to Darren Obbard, while those of *D. erecta* are reproduced from Yassin et al. (2016) under Creative Commons licence 4.0.

We begin the inference with PoMoSelect to determine the tree and the level of gBGC in *Drosophila* subspecies. We analyze $t_{\mathrm{MSE}}$ region in *Drosophila erecta* dark and light as well as *santomea, yakuba, melanogaster*, and *simulans*. The tree topology obtained with PoMoSelect, as shown in Fig. 7 (left), closely resembles the topology obtained by Yassin et al. (2016) using the multispecies coalescent method.

**Fig. 8.**
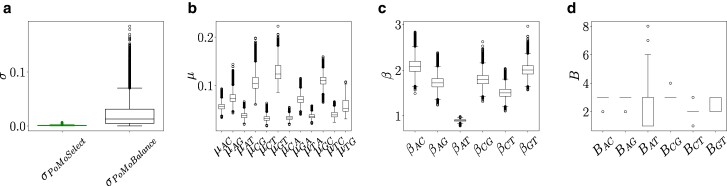
Posterior distributions derived from experimental data extracted from the $t_{\mathrm{MSE}}$ region of six subspecies, as shown in Fig. 7 for PoMoSelect inference, and four *Drosophila* subspecies, namely *D. erecta* dark and light, *melanogaster*, and *simulans* for PoMoBalance inference. The corresponding SFS for the PoMobalance is presented in Fig. 9. a) Estimated rates of gBGC with PoMoSelect on the left and PoMoBalance on the right. b) Mutation rates, c) strength of BS, and d) preferred frequencies for BS, all inferred using PoMoBalance.

The gBGC rate $\sigma_{\mathrm{Sel}}$, inferred with PoMoSelect alongside the tree in Fig. 7 (right), is shown in Fig. 8a with box plot on the left, and it is quite low, as observed in experiments (Robinson et al. 2014). Refer to supplementary Table S3, Supplementary Material online, for the inferred parameters and effective sample sizes (ESS). The rest of the box plots in Fig. 8 show the posterior distributions of the parameters inferred with PoMoBalance for four *Drosophila* subspecies, namely *D. erecta* dark and light, *melanogaster*, and *simulans*. Here we discard sequences of *D. santomea* and *yakuba* since they introduce noise into BS detection due to low numbers of individuals in the dataset, while still acceptable for PoMoSelect analysis. The results for all subspecies are presented in the supplementary Figs. S3 and S4, Supplementary Material online.

**Fig. 9.**
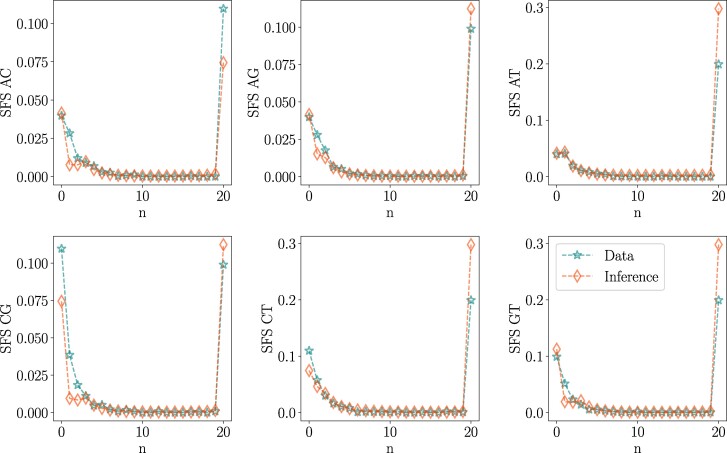
SFS representation for the $t_{\mathrm{MSE}}$ region corresponding to the PoMoBalance analysis in Fig. 8 for four subspecies of *Drosophila*, depicted in stars, compared with the inferred SFS indicated by diamonds.

The posterior distribution for $\sigma_{\mathrm{PoMoBalance}}$ in Fig. 8a, inferred with PoMoBalance, is much wider than those for $\sigma_{\mathrm{PoMoSelect}}$ as it is challenging to detect GC-bias and BS simultaneously. Thus, we advocate a mixed approach by running PoMoSelect and PoMoBalance in parallel to get more accurate estimates. For example, we learn the tree topology from PoMoSelect and then fix the estimated topology for PoMoBalance analysis. The mutation rates in Fig. 8b show great convergence and ESS > 200 for all MCMC chains. The presence of BS is detected in most of the spectra, indicated by β>1 in Fig. 8c, while for $\beta_{AT}$, we observe purging of selection, indicated by β<1. The preferred frequencies in Fig. 8d coincide or are not far away from the positions of BS peaks in the experimental SFS as shown in Fig. 9.

We performed all analyses using the UK Crop Diversity: Bioinformatics high-performance computing (HPC) Resource and the parallel implementation of RevBayes with 24 parallel processes. The computational time was 85 h for PoMoSelect (6 subspecies, each containing 6–25 individuals) and 118 h (4 subspecies, each containing 6–25 individuals) for PoMoBalance to analyze the $t_{\mathrm{MSE}}$ region. For comparison, multispecies coalescent analysis for two species with introgression but without BS would take 5 days (Flouri et al. 2020).

## Discussion

Our study validated the implementations of PoMoSelect and PoMoBalance through simulation-based calibration in section “Validation Analysis for PoMoSelect and PoMoBalance”. Additionally, we conducted a diverse set of tests using data generated from both our custom simulator, based on the Moran model, and the evolutionary simulation framework SLiM in section “Modelling the BS with PoMoBalance” (Haller and Messer 2019). The PoMos demonstrated notable adaptability, particularly in the context of inferring data simulated via SLiM, which incorporates more complex evolutionary dynamics than the Moran model.

While SLiM, grounded in the Wright–Fisher model, shares similarities with the Moran model, it introduces additional complexities such as genetic recombination, population demography (changes in population sizes), and diploid organisms with intricate interactions between drift and heterozygote advantage. Despite these challenges, PoMoBalance performs well in locating BS polymorphic peaks. To align SLiM diploids with PoMos, we treated them as two haplotypes in PoMos.

Notably, while overestimating the GC-bias rate, PoMoBalance excelled in identifying preferred frequencies, specifically in the middle of the SFS, corresponding to heterozygote advantage in SLiM. This represents a unique advantage compared to previous methods, which, while suggestive of the presence of BS, cannot pinpoint specific combinations of alleles, strengths, and preferred frequencies of BS. It is important to acknowledge potential correlations between *β* and *σ*, which limits their inference. To address this, we advocate for incorporating extra moves into the MCMC, as discussed in section “Bayesian Inference Using PoMoBalance with RevBayes”. The comparative analysis with MuteBaSS and MULLET indicates that our method demonstrates the strongest evidence of BS for data involving the heterozygote advantage. However, this result must be interpreted with caution since we assess the performance of our method using the BF approach, while we derive averaged statistics for the other methods (see supplementary material 4, Supplementary Material online).

In section “Detection of BS in *Drosophila erecta*”, we applied PoMoSelect and PoMoBalance to analyze experimental genomic data from *Drosophila erecta*, specifically focusing on the $t_{\mathrm{MSE}}$ region known to exhibit BS (Yassin et al. 2016). Our application of PoMos reproduced previous insights by Yassin et al. (2016) into the phylogenetic relationships among *Drosophila* subspecies.

Note that the outcomes of the inference for CG-bias rate and mutation rates are presented in terms of the virtual PoMos population sizes, which typically differ from the actual population sizes. To accurately reflect the actual population dynamics in *Drosophila*, it is necessary to map the values of *μ*, *σ*, *β*, and *B* from virtual PoMos size to effective population size (see supplementary material 2, Supplementary Material online). This mapping results in substantially reduced parameter values for *σ* and *μ*, as found by Borges et al. (2019), given the large effective population sizes characteristic of *Drosophila* (Kelley et al. 2005). The mapping for the preferred frequency is relatively straightforward, and we plan to propose a mapping for the BS strengths and the nonreversible coefficients in future research.

Through PoMoBalance analysis, we detect BS in the majority of allele combinations, in contrast to the absence of BS peaks in neutral regions. Additionally, we observe the purging of selection for AT alleles, signifying the removal of polymorphisms at a rate higher than expected under neutral conditions. While this discovery showcases the flexibility of our method, interpreting its biological implications is challenging. Moreover, such interpretation might be unnecessary, as the mean value for $\beta_{AT}$ is only slightly smaller than 1, indicating neutrality expectations and suggesting a relatively weak effect.

## Conclusion

We incorporated the PoMoBalance model, a generalized form of PoMos capable of detecting BS, into RevBayes, a widely used phylogenetic software based on Bayesian inference. This integration enriches the resources available to researchers engaged in phylogenetic analysis, providing a robust framework for precise species tree inference and concurrent parameter estimation. Notably, our implementation allows for the estimation of BS, including preferred frequencies and specific alleles under selection, while also disentangling it from other forms of selection. PoMoBalance exhibits versatility in capturing various selection types, including purging selection, observed when the level of observed polymorphisms is lower than expected via genetic drift and DS. These effects may arise from a combination of dominance effects, such as underdominance, or purifying selection in the context of background selection, etc.

In general, we provide a comprehensive framework to use PoMos for the estimation of phylogenetic trees, GC-bias, and BS. The approach involves several key steps. First, we employ the PoMoSelect to estimate tree topology, GC-bias rate, and mutations. Subsequently, we use PoMoBalance to estimate all parameters, allowing branch lengths to vary while maintaining a fixed topology learned from PoMoSelect. It is worthwhile to validate the results by comparing the inferred values with PoMoBalance estimates that include a fixed GC-bias rate learned from PoMoSelect. The selection of the best candidates is based on the agreement between the inferred SFS and that estimated from the data. Lastly, in this framework, PoMoBalance is selectively applied to regions that are likely under BS, such as the MHC locus in *Homo sapiens*.

The adaptability and versatility of PoMos address a need in the analysis of complex genomic datasets since our framework provides accurate phylogenetic inferences across multiple timescales and demonstrate potential for application in genome-wide scans through the parallel inference of multiple genomic regions. The other benefit of PoMos is scalability in terms of the number of species; it is capable of handling dozens of species (Borges et al. 2022b). In future, we aim to investigate additional genomic factors intertwined with BS, with a specific focus on exploring the role of linkage disequilibrium and its impact on the detection of BS.

## Software Availability

The software RevBayes (Höhna et al. 2016, 2017, 2018) is available at https://revbayes.github.io/. PoMoBalance tutorial at https://revbayes.github.io/tutorials/pomobalance/.

## Supplementary Material

msae138_Supplementary_Data

## Data Availability

The data and the code for PoMoBalance analysis concerning simulation-based calibration, Moran simulator, and SLiM are available via GitHub (https://github.com/sb2g14/PoMoBalance). The sequencing data for *Drosophila erecta* and *orena* used in the analysis were previously published by Yassin et al. (2016), and the data for multiple individuals of other related subspecies of *Drosophila* were obtained via NCBI (National Center for Biotechnology Information) BLAST (**B**asic **L**ocal **A**lignment **S**earch **T**ool) (https://blast.ncbi.nlm.nih.gov/Blast.cgi).
