# Supplementary material for: Polymorphism-Aware Models in RevBayes: Species Trees, Disentangling Balancing Selection, and GC-Biased Gene Conversion
Source: Mol Biol Evol. 2024 Jul 9;41(7):msae138. doi: 10.1093/molbev/msae138 (PMC11272101; doi:10.1093/molbev/msae138)
Supplement: msae138_Supplementary_Data [file msae138_supplementary_data.pdf]

Supplementary Material 1 Stationary Distribution and Reversibility of PoMoBalance

The stationary distribution ψ provides the opportunity to investigate the long-term behavior of the interplay between mutational bias, genetic drift, directional, and balancing selection on population diversity. In the biallelic case, the Moran dynamic exemplifies a birth-and-death process, known for its reversibility. Consequently, we obtained the stationary distribution by initially formulating the detailed balance equations. To simplify the notation, we redefine the state $\{na_i, (N-n)a_j\}$ to only represent the frequency of the a_i allele: i.e., $\{n\}$.

$$\psi_{\{n\}} q^{\{na_i,(N-n)a_j\} \to \{(n+1)a_i,(N-n-1)a_j\}} = \psi_{\{n+1\}} q^{\{(n+1)a_i,(N-n-1)a_j\} \to \{na_i,(N-n)a_j\}} \quad . \tag{S1}$$

The detailed balance equations allow us to derive the following recursive formula, which is employed to obtain the stationary quantities for both fixed and polymorphic states

$$\psi_{\{n\}} = \psi_{\{0\}} \prod_{k=1}^{n} \frac{q^{\{k-1\} \to \{k\}}}{q^{\{k\} \to \{k-1\}}} \quad . \tag{S2}$$

If we set $n = N$, the recursive formula becomes

$$\psi_{\{N\}} = \psi_{\{0\}} \prod_{k=1}^{N} \frac{q^{\{k-1\} \to \{k\}}}{q^{\{k\} \to \{k-1\}}} = \psi_{\{0\}} \frac{q^{\{0\} \to \{1\}}}{q^{\{1\} \to \{0\}}} \cdots \frac{q^{\{n-1\} \to \{n\}}}{q^{\{n\} \to \{n-1\}}} \cdots \frac{q^{\{N-1\} \to \{N\}}}{q^{\{N\} \to \{N-1\}}} \quad , \tag{S3}$$

from which by considering the rates of the process defined in the rate matrix Q in Equation (2), we find the normalized stationary quantities for the fixed states

$$\frac{\psi_{\{N\}}}{\psi_{\{0\}}} = \frac{\mu_{a_j a_i}}{\mu_{a_i a_j}} \frac{(1+\sigma_{a_i})^{N-1}}{(1+\sigma_{a_j})^{N-1}} \beta^{2B_{a_i a_j} - N} \quad . \tag{S4}$$

An interesting aspect is that the differentiated impact of BS in the fixed states disappears when the balanced frequency sits in the middle of the frequency spectrum (i.e., $B_{a_i a_j} = N/2$). By applying the Kolmogorov criterion to each closed chain in the PoMoBalance model described with Equation (2) we ensure that reversibility is satisfied when $B_{a_i a_j} = N/2$ and breaks in all other cases.

The stationary distributions $\psi_{\{n\}}$ may be multiplied by any arbitrary constant without affecting

the final result thanks to the normalisation condition. Thus, we are safe to assume that we could set

$$\psi_{\{0\}} = k^{-1}\mu_{a_i a_j}(1+\sigma_{a_j})^{N-1} \quad , \tag{S5}$$

where k is obtained from the normalisation condition $\sum_{i=0}^{N}\psi_{\{i\}} = 1$. Then from equation (S4) we find

$$\psi_{\{N\}} = k^{-1}\mu_{a_j a_i}(1+\sigma_{a_i})^{N-1}\beta^{2B_{a_i a_j}-N} \quad . \tag{S6}$$

Similarly to the fixed states, the stationary measures for the polymorphic states can be derived using the recursive formula in equation (S2)

$$\begin{aligned}\psi_{\{n\}} &= \psi_{\{0\}}\frac{q^{\{0\}\to\{1\}}}{q^{\{1\}\to\{0\}}}\cdots\frac{q^{\{k\}\to\{k+1\}}}{q^{\{k\}\to\{k-1\}}}\cdots\frac{q^{\{n-1\}\to\{n\}}}{q^{\{n\}\to\{n-1\}}} \\ &= k^{-1}\mu_{a_j a_i}\mu_{a_i a_j}\frac{N}{n(N-n)}(1+\sigma_{a_j})^{N-n-1}(1+\sigma_{a_i})^{n-1}\beta^{B_{a_i a_j}-|n-B_{a_i a_j}|-1} \quad .\end{aligned} \tag{S7}$$

This solution clearly illustrates the contribution of mutational bias, genetic drift, directional selection, and BS to the frequency of polymorphic states. As expected, the BS term is highest when $n = B_{a_i a_j}$ and decays in the direction of the boundary states. This feature becomes evident when we compare the stationary distribution with and without the effect of BS in Figure S1.

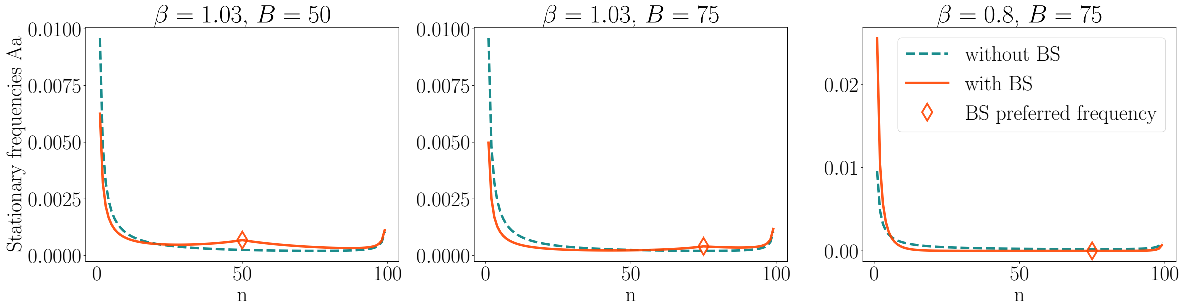

Figure S1: The plots depict the stationary distribution of a population of $N = 100$ individuals, and a biallelic locus with alleles A and a that evolves under under mutational bias ($\mu_{Aa} = 0.02 > \mu_{aA} = 0.01$), directional selection ($\sigma_A = 0.01 > \sigma_a = 0.0$) and three regimes of BS. Here we present frequencies in the range $[1, N-1]$ to avoid very high tails that dominate the BS peak.

Because we are interested in modelling BS, we have been assuming that $\beta_{a_i a_j}$ acts to maintain diversity at a certain frequency $B_{a_i a_j}$. Mathematically speaking, we have been assuming that $\beta_{a_i a_j} > 1$. However, an interesting behaviour emerges when $\beta_{a_i a_j} < 1$. In this case, the BS term acts to purge variation more than what is already expected by genetic drift and directional selection, as shown in Figure S1. We refer to this regime as purging selection.

We normalize the obtained stationary quantities obtained in equations (S5), (S6) and (S7) to sum up to 1. The stationary distribution normalization constant is

$$k = \mu_{a_i a_j}(1+\sigma_{a_j})^{N-1} + \mu_{a_j a_i}\mu_{a_i a_j}\sum_{n=1}^{N-1}\frac{N}{n(N-n)}(1+\sigma_{a_j})^{N-n-1}(1+\sigma_{a_i})^{n-1}\beta_{a_i a_j}^{B_{a_i a_j}-|n-B_{a_i a_j}|-1}$$
$$+ \mu_{a_j a_i}(1+\sigma_{a_i})^{N-1}\beta^{2B_{a_i a_j}-N} \quad . \tag{S8}$$

Supplementary Material 2 Virtual population size in PoMoBalance

In PoMos, we operate with virtual population sizes for computational efficiency. Although the effective population size does not directly reflect the exact mutation rates, gBGC coefficients, and balancing selection coefficients, it provides a means to assess the relative strength of these effects. Mapping these coefficients to actual population sizes is feasible but depends on the specific PoMos utilised in the analysis.

For instance, Borges *et al.* (2019) detailed in Appendix A the mapping between the virtual sizes of reversible PoMoSelect for two populations, A and A' with respective sizes N and M. In the case of reversible PoMoBalance, the scaling would be similar, utilising SFS in Equations (S5), (S6) and (S7) where σ and β would need to be scaled together. The preferred frequency scales as $\frac{B}{N} = \frac{B'}{M}$, where B represents the frequency of population A, and B' is the frequency of population A'.

Regarding non-reversible PoMos, the mapping of the preferred frequency remains unchanged, while the other coefficients require numerical mapping of solutions $P(t) = \exp(tQ)$ for population A and $P'(t) = \exp(tQ')$ for A'. This aspect requires further investigation in future research endeavors.

Supplementary Material 3 Simulations with SLiM

The original scripts for SLiM simulations can be found in the supplementary repository (https://github.com/sb2g14/PoMoBalance). We ran nucleotide models, simulating 10^5 genomic sites with drift only using `initializeMutationTypeNuc("m1", 0.5, "f", 0.0)`, drift+gc-bias using `initializeGeneConversion(0.3, 1500, 0.80, 1.0)`, drift+heterozygote advantage with `initializeMutationTypeNuc("m2", 1.1, "f", 0.1)`, where the coefficient 1.1 simulates overdominance. We set the mutation rate to 10^{-6} and the recombination rate to 10^{-5}. These rates are higher than physiological ones for computational purposes, but they work well for the purposes of our analysis.

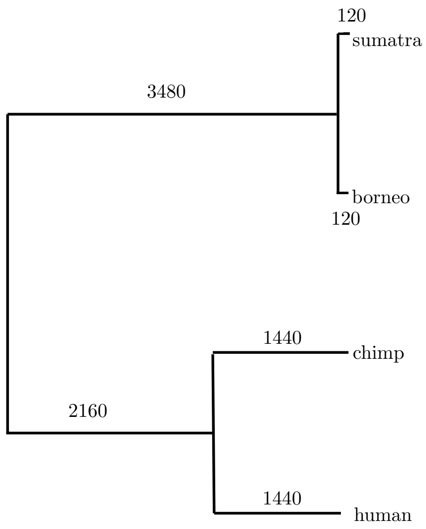

Figure S2: Phylogenetic tree simulated with SLiM, the inferred tree is presented in Figure 4 (C), here the branch lengths are expressed in the simulated generations.

We initialised a population of 2000 individuals of *Homininae* and evolved them for 10000 populations as a burn-in step. Then we split them into 1000 of *Hominini* and 1000 of *Gorillini*. Following the numbers of generations shown in Figure 1 we end up with *Orangutan sumatra*, *Orangutan borneo*, *chimp* and *human*, each containing 500 individuals.

Finally, the ancestral sequences (`.FASTA`) and polymorphic data (`.VCF`) are written out as output for each population.

Note that in the inference with the data simulated with SLiM, as shown in Table S2, the BS strengths β for the neutral case and GC-biased case are significantly underestimated (the most are 0.7 instead of 1). This is presumably due to noise in the simulations, especially when SLiM diploids are mapped to small population sizes in PoMos. Interestingly, with an increase in the

population size, such misspecification is reduced, and for $N = 20$, we obtain $\beta = 0.9$.

Supplementary Material 4 Model comparison

In Table 4 we strive to compare several approaches for detecting BS. Here, we utilise data generated with SLiM to produce Figure 6 For MuteBaSS ($\mathrm{HKA_{trans}}$, NCD, $\mathrm{NCD_{opt}}$, $\mathrm{NCD_{sub}}$) we determine the optimal window size from the sliding window analysis, which yields corresponding scores for each window. We consider a range of window sizes (refer to Table S4 for drift, Table S5 for gBGC and Table S6 for BS) with a small step size (10 bases) to ensure a sufficient number of scores (s_i^{reg}) in each run. in each run. The resulting scores, presented in Tables S4, S5 and S6, represent averages of all s_i^{reg} within each run:

$$s^{\mathrm{reg}} = \sum_{i=1}^{M} s_i^{\mathrm{reg}}, \tag{S9}$$

where M is the total number of calculated scores in each sliding window run, and reg denotes the regime under which the data was obtained (drift, gBGC, or BS). The scaled scores provided in Table 4 are calculated by scaling each averaged score by the neutral case $\|s^{\mathrm{reg}}\| = \frac{s^{\mathrm{reg}}}{s^{\mathrm{drift}}}$. The optimal window size is selected from all scores in Table S5 where $\|s^{\mathrm{gBGC}}\|$ is closest to 1 (except for $\mathrm{HKA_{trans}}$, which is the only one testing for selection), indicating no BS in the data generated with gBGC, while $\|s^{\mathrm{BS}}\|$ simultaneously reaches its maximum.

The scaled scores for MULLET ($\mathrm{T_{1trans}}$ and $\mathrm{T_{2trans}}$) (Cheng and DeGiorgio, 2019) are calculated similarly to MuteBaSS, with the only difference being the omission of the step size. In the T statistics, the region assessed at each step centers on a test-informative site and includes a set number of informative sites both upstream and downstream, rather than employing a fixed physical window size based on the number of nucleotides. It's worth noting that for a large number of informative sites (over 500 or, in some cases, 1000), the T statistics always returns NAN.

The log Bayes Factors in PoMos analysis are calculated from the difference of marginal log-likelihoods in each regime

$$\log(BF^{\mathrm{reg}}) = \log \mathbb{P}(\mathbf{X}^{\mathrm{reg}} \mid M_1) - \log \mathbb{P}(\mathbf{X}^{\mathrm{reg}} \mid M_0), \tag{S10}$$

where M_0 represents the PoMoSelect model, while M_1 represents PoMoBalance. $\mathbf{X}^{\text{reg}}$ is the data generated in certain regime (drif, gBGC and BS). $\log \mathbb{P}(\mathbf{X}^{\text{reg}} \mid M_0)$ and $\log \mathbb{P}(\mathbf{X}^{\text{reg}} \mid M_1)$ are corresponding log-likelihoods. In general, $\log(BF^{\text{reg}})$ explains how much PoMoBalance explains data better comparing to PoMoSelect. The values for log-likelihoods in our analysis are $\log \mathbb{P}(\mathbf{X}^{\text{drift}} \mid M_0) = -290451.8918$, $\log \mathbb{P}(\mathbf{X}^{\text{gBGC}} \mid M_0) = -282710.9797$, $\log \mathbb{P}(\mathbf{X}^{\text{ BS}} \mid M_0) = -308840.628$, $\log \mathbb{P}(\mathbf{X}^{\text{drift}} \mid M_1) = -289601.7246$, $\log \mathbb{P}(\mathbf{X}^{\text{gBGC}} \mid M_1) = -281708.4163$ and $\log \mathbb{P}(\mathbf{X}^{\text{ BS}} \mid M_1) = -245036.4488$. Similarly to the previous cases the scaled score is $\| \log(BF^{\text{reg}}) \| = \frac{\log(BF^{\text{reg}})}{\log(BF^{\text{drift}})}$.

Supplementary Material 5 Supplementary Figures

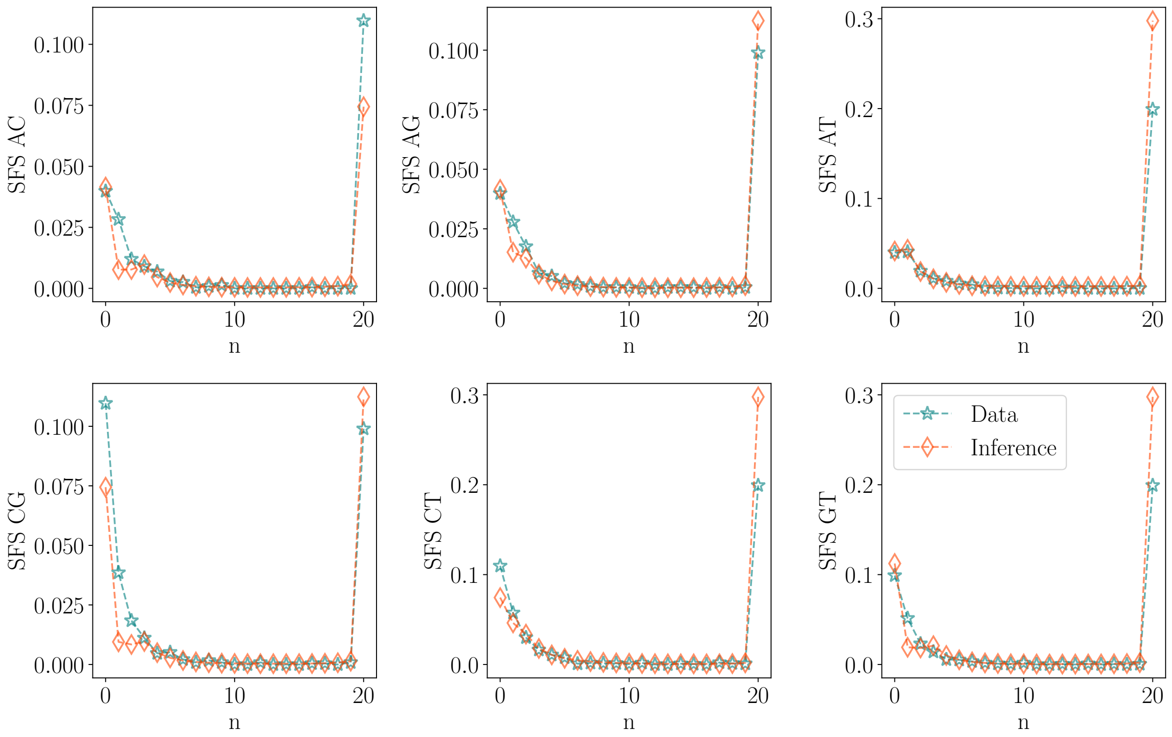

Figure S3: SFS representation for the t_{MSE} region in six subspecies of *Drosophila*, denoted by blue stars, is compared with the SFS inferred using PoMoBalance, represented by red diamonds.

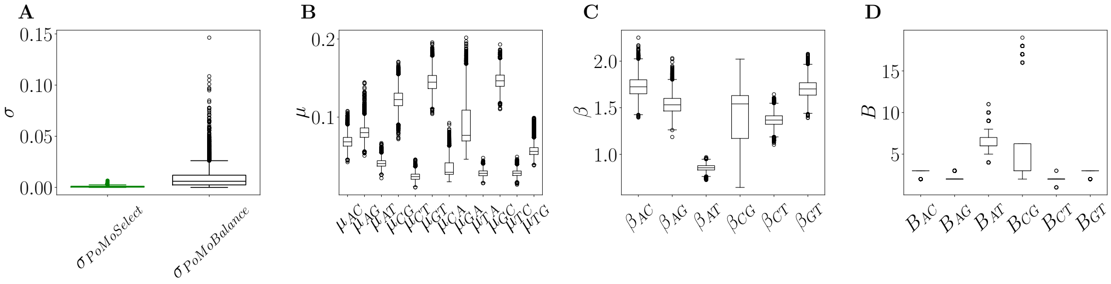

Figure S4: Posterior distributions derived from experimental data extracted from the t_{MSE} region of six *Drosophila* subspecies. The corresponding tree and SFS are presented in Figure 7 and S3. (A) Estimated rates of gBGC with PoMoSelect in green and PoMoBalance in black. (B) Mutation rates, (C) strength of BS and (D) preferred frequencies for BS, all inferred using PoMoBalance.

Supplementary Material 6 Supplementary Tables

	Drift			GC-bias		
Variable	True values	Posterior mean	95 % credible interval	True values	Posterior mean	95 % credible interval
sigma	0	0.042	[0, 0.205]	0.1	0.098	[0.0013, 0.197]
pi_A	0.25	0.257	[0.253, 0.261]	0.25	0.249	[0.244, 0.255]
pi_C	0.25	0.252	[0.247, 0.256]	0.25	0.254	[0.248, 0.259]
pi_G	0.25	0.242	[0.238, 0.247]	0.25	0.249	[0.244, 0.254]
pi_T	0.25	0.249	[0.244, 0.254]	0.25	0.248	[0.243, 0.253]
rho_AC	0.1	0.098	[0.095, 0.1]	0.1	0.099	[0.096, 0.101]
rho_AG	0.1	0.1	[0.098, 0.103]	0.1	0.1	[0.097, 0.103]
rho_AT	0.1	0.098	[0.096, 0.101]	0.1	0.1	[0.097, 0.103]
rho_CG	0.1	0.102	[0.099, 0.105]	0.1	0.1	[0.097, 0.103]
rho_CT	0.1	0.099	[0.097, 0.102]	0.1	0.098	[0.095, 0.1]
rho_GT	0.1	0.101	[0.098, 0.103]	0.1	0.102	[0.1, 0.105]
beta_AC	1	1.039	[0.968, 1.198]	1	1.014	[0.911, 1.098]
beta_AG	1	1.01	[0.876, 1.198]	1	1.018	[0.926, 1.113]
beta_AT	1	1.006	[0.976, 1.035]	1	0.998	[0.959, 1.042]
beta_CG	1	0.978	[0.945, 1.03]	1	0.997	[0.969, 1.025]
beta_CT	1	1.003	[0.807, 1.147]	1	0.999	[0.892, 1.077]
beta_GT	1	1.029	[0.959, 1.232]	1	0.991	[0.894, 1.072]
	**BS**			**GC-bias + BS**		
sigma	0	0.0014	[0, 0.004]	0.1	0.0014	[0, 0.004]
pi_A	0.25	0.248	[0.242, 0.253]	0.25	0.254	[0.247, 0.26]
pi_C	0.25	0.254	[0.249, 0.258]	0.25	0.252	[0.247, 0.258]
pi_G	0.25	0.245	[0.242, 0.249]	0.25	0.247	[0.243, 0.252]
pi_T	0.25	0.253	[0.25, 0.257]	0.25	0.248	[0.243, 0.252]
rho_AC	0.1	0.102	[0.099, 0.106]	0.1	0.098	[0.094, 0.101]
rho_AG	0.1	0.101	[0.099, 0.105]	0.1	0.102	[0.099, 0.105]
rho_AT	0.1	0.101	[0.098, 0.104]	0.1	0.099	[0.095, 0.102]
rho_CG	0.1	0.1	[0.097, 0.102]	0.1	0.1	[0.097, 0.103]
rho_CT	0.1	0.098	[0.095, 0.1]	0.1	0.099	[0.096, 0.101]
rho_GT	0.1	0.101	[0.099, 0.103]	0.1	0.098	[0.096, 0.1]
B_AC	2	2	[2.0, 2.0]	2	2	[2.0, 2.0]
B_AG	2	2	[2.0, 2.0]	2	2	[2.0, 2.0]
B_AT	2	2	[2.0, 2.0]	2	2	[2.0, 2.0]
B_CG	2	2	[2.0, 2.0]	2	2	[2.0, 2.0]
B_CT	2	2	[2.0, 2.0]	2	2	[2.0, 2.0]
B_GT	2	2	[2.0, 2.0]	2	2	[2.0, 2.0]
beta_AC	2	1.991	[1.934, 2.047]	2	1.991	[1.934, 2.047]
beta_AG	2	1.982	[1.931, 2.029]	2	1.982	[1.931, 2.029]
beta_AT	2	1.994	[1.958, 2.029]	2	1.994	[1.958, 2.029]
beta_CG	2	2.067	[2.022, 2.117]	2	2.067	[2.022, 2.117]
beta_CT	2	2.013	[1.98, 2.043]	2	2.013	[1.98, 2.043]
beta_GT	2	1.969	[1.939, 1.996]	2	1.969	[1.939, 1.996]

Table S1: Results of converged MCMC chain for Figure 4 (A), (B) and 6 (A), (B), (C).

	Drift			GC-bias		
Variable	True values	Posterior mean	95 % credible interval	True values	Posterior mean	95 % credible interval
sigma	0	0.0026	[0, 0.0058]	0.04	0.032	[0.023, 0.042]
pi_A	0.25	0.252	[0.248, 0.257]	0.25	0.166	[0.16, 0.172]
pi_C	0.25	0.249	[0.245, 0.253]	0.25	0.23	[0.224, 0.238]
pi_G	0.25	0.248	[0.244, 0.252]	0.25	0.302	[0.294, 0.311]
pi_T	0.25	0.251	[0.246, 0.255]	0.25	0.301	[0.291, 0.311]
rho_AC	0.04	0.041	[0.04, 0.043]	0.03	0.047	[0.044, 0.049]
rho_AG	0.04	0.042	[0.041, 0.044]	0.03	0.037	[0.035, 0.038]
rho_AT	0.04	0.041	[0.039, 0.043]	0.03	0.031	[0.029, 0.032]
rho_CG	0.04	0.042	[0.04, 0.044]	0.03	0.031	[0.03, 0.033]
rho_CT	0.04	0.039	[0.038, 0.041]	0.03	0.029	[0.028, 0.031]
rho_GT	0.04	0.039	[0.037, 0.04]	0.03	0.03	[0.028, 0.031]
beta_AC	1	0.71	[0.692, 0.727]	1	0.749	[0.73, 0.769]
beta_AG	1	0.719	[0.7, 0.736]	1	0.822	[0.799, 0.845]
beta_AT	1	0.714	[0.696, 0.732]	1	1.094	[1.069, 1.12]
beta_CG	1	0.7	[0.682, 0.717]	1	0.794	[0.775, 0.814]
beta_CT	1	0.711	[0.693, 0.73]	1	0.849	[0.815, 0.883]
beta_GT	1	0.733	[0.714, 0.751]	1	0.694	[0.668, 0.718]
	**BS**					
sigma	0	0.017	[0.01, 0.025]			
pi_A	0.25	0.268	[0.259, 0.276]			
pi_C	0.25	0.233	[0.224, 0.241]			
pi_G	0.25	0.231	[0.222, 0.238]			
pi_T	0.25	0.269	[0.261, 0.277]			
rho_AC	0.00032	0.00031	[0.00029, 0.00034]			
rho_AG	0.00032	0.00032	[0.0003, 0.00034]			
rho_AT	0.00032	0.0003	[0.00027, 0.00032]			
rho_CG	0.00032	0.00033	[0.0003, 0.00036]			
rho_CT	0.00032	0.00029	[0.00027, 0.00031]			
rho_GT	0.00032	0.00029	[0.00027, 0.00032]			
B_AC	5.0	5.0	[5.0, 5.0]			
B_AG	5.0	5.0	[5.0, 5.0]			
B_AT	5.0	5.0	[5.0, 5.0]			
B_CG	5.0	5.0	[5.0, 5.0]			
B_CT	5.0	5.0	[5.0, 5.0]			
B_GT	5.0	5.0	[5.0, 5.0]			
beta_AC	7.0	6.956	[6.814, 7.085]			
beta_AG	7.0	6.958	[6.827, 7.095]			
beta_AT	7.0	6.981	[6.839, 7.107]			
beta_CG	7.0	7.093	[6.956, 7.228]			
beta_CT	7.0	7.158	[7.027, 7.304]			
beta_GT	7.0	7.074	[6.934, 7.217]			

Table S2: Results of converged MCMC chain for Figure 4 (C), (D) and 6 (D), (E), (F).

	**PoMoSelect**			**PoMoBalance**		
Variable	Posterior mean	95 % credible interval	ESS	Posterior mean	95 % credible interval	ESS
sigma	0.00081	[0, 0.0023]	7660	0.022	[0, 0.074]	472
pi_A	0.176	[0.139, 0.216]	1773	0.422	[0.358, 0.488]	341
pi_C	0.292	[0.254, 0.33]	3696	0.32	[0.278, 0.365]	1645
pi_G	0.225	[0.191, 0.258]	3760	0.151	[0.115, 0.193]	241
pi_T	0.306	[0.272, 0.343]	4787	0.107	[0.082, 0.134]	641
rho_AC	0.028	[0.021, 0.036]	2950	0.178	[0.135, 0.226]	759
rho_AG	0.032	[0.024, 0.041]	3008	0.255	[0.193, 0.331]	2654
rho_AT	0.025	[0.019, 0.031]	2267	0.304	[0.238, 0.379]	1920
rho_CG	0.022	[0.018, 0.026]	4943	0.224	[0.173, 0.28]	1286
rho_CT	0.02	[0.017, 0.023]	4726	0.344	[0.284, 0.409]	2377
rho_GT	0.018	[0.015, 0.021]	4042	0.379	[0.286, 0.485]	406
B_AC	-	-	-	2.995	[3.0, 3.0]	2375
B_AG	-	-	-	2.86	[2.0, 3.0]	1315
B_AT	-	-	-	1.954	[1.0, 4.0]	386
B_CG	-	-	-	3	[3.0, 3.0]	29347
B_CT	-	-	-	2.016	[2.0, 2.0]	6304
B_GT	-	-	-	2.638	[2.0, 3.0]	165
beta_AC	-	-	-	2.091	[1.782, 2.417]	3515
beta_AG	-	-	-	1.73	[1.47, 2.009]	1166
beta_AT	-	-	-	0.898	[0.854, 0.941]	1646
beta_CG	-	-	-	1.796	[1.526, 2.067]	4819
beta_CT	-	-	-	1.508	[1.291, 1.73]	559
beta_GT	-	-	-	2.011	[1.752, 2.283]	9919

Table S3: Results of the inference combined from 4 MCMC chains for Figure 8 and 9.

Score<br>Window size	HKA_{trans}	NCD	NCD_{opt}	NCD_{sub}	T_{1trans}	T_{2trans}
10	-0.875	0.492	0.294	0.463	-7.851	-23.954
50	-0.871	0.492	0.294	0.464	-47.773	-127.0
100	-0.793	0.492	0.294	0.464	-113.0	-256.0
200	-0.729	0.492	0.294	0.464	-276.0	-526.0
500	-0.667	0.492	0.294	0.464	-873.0	-
700	-0.702	0.492	0.294	0.464	-1278.0	-
1000	-0.711	0.492	0.294	0.464	-	-
2000	-0.526	0.492	0.294	0.464	-	-
10000	0.793	0.492	0.294	0.464	-	-
20000	1.573	0.492	0.294	0.464	-	-
30000	2.37	0.492	0.294	0.464	-	-
40000	2.647	0.492	0.294	0.464	-	-
50000	2.805	0.492	0.294	0.464	-	-
60000	3.837	0.492	0.294	0.464	-	-
70000	3.418	0.492	0.294	0.464	-	-
80000	2.574	0.492	0.294	0.464	-	-
90000	0.604	0.492	0.294	0.464	-	-
100000	-3.5e-06	0.493	0.294	0.464	-	-

Table S4: Tests run with MuteBaSS (HKA_{trans}, NCD, NCD_{opt}, NCD_{sub}) and MULLET (T_{1trans}, T_{2trans}) (Cheng and DeGiorgio, 2019), obtained by averaging the scores with various window sizes and a step size of 10 nucleotides. The data were generated with SLiM on the tree shown in Figure 4 (C) under neutral (drift) conditions.

Score<br>Window size	HKA_{trans}	NCD	NCD_{opt}	NCD_{sub}	T_{1trans}	T_{2trans}
10	-0.813	0.493	0.295	0.464	-8.103	-23.485
50	-0.661	0.493	0.295	0.466	-48.246	-123.0
100	-0.479	0.493	0.295	0.466	-114.0	-249.0
200	-0.361	0.493	0.295	0.466	-280.0	-495.0
500	-0.297	0.493	0.295	0.466	-	-
700	-0.249	0.493	0.295	0.466	-	-
1000	-0.21	0.493	0.295	0.466	-	-
2000	-0.127	0.493	0.295	0.466	-	-
10000	0.722	0.493	0.295	0.466	-	-
20000	1.01	0.493	0.295	0.466	-	-
30000	0.52	0.493	0.295	0.466	-	-
40000	0.154	0.493	0.295	0.466	-	-
50000	-0.07	0.493	0.295	0.466	-	-
60000	-0.77	0.494	0.295	0.466	-	-
70000	-0.425	0.493	0.295	0.466	-	-
80000	0.014	0.493	0.295	0.466	-	-
90000	0.042	0.493	0.295	0.466	-	-
100000	0.00072	0.493	0.295	0.466	-	-

Table S5: Tests run with MuteBaSS (HKA_{trans}, NCD, NCD_{opt}, NCD_{sub}) and MULLET (T_{1trans}, T_{2trans}) (Cheng and DeGiorgio, 2019), obtained by averaging the scores with various window sizes and a step size of 10 nucleotides. The data were generated with SLiM on the tree shown in Figure 4 (C) under gBGC.

Score<br>Window size	HKA_{trans}	NCD	NCD_{opt}	NCD_{sub}	T_{1trans}	T_{2trans}
10	-0.824	0.493	0.298	0.49	-15.954	-56.416
50	-1.2	0.494	0.298	0.49	-108.0	-406.0
100	-1.572	0.494	0.298	0.49	-249.0	-986.0
200	-1.563	0.494	0.298	0.49	-557.0	-2336.0
500	-1.957	0.494	0.298	0.49	-1541.0	-6814.0
700	-1.633	0.494	0.298	0.49	-2213.0	-9779.0
1000	-1.104	0.494	0.298	0.49	-3238.0	-
2000	-0.422	0.494	0.298	0.49	-	-
10000	0.705	0.494	0.298	0.49	-	-
20000	1.573	0.494	0.298	0.49	-	-
30000	2.418	0.494	0.298	0.49	-	-
40000	3.28	0.494	0.298	0.49	-	-
50000	4.184	0.494	0.298	0.49	-	-
60000	5.114	0.494	0.298	0.49	-	-
70000	6.029	0.494	0.298	0.49	-	-
80000	6.91	0.494	0.298	0.49	-	-
90000	7.658	0.494	0.298	0.49	-	-
100000	-7.6e-08	0.494	0.298	0.49	-	-

Table S6: Tests run with MuteBaSS (HKA_{trans}, NCD, NCD_{opt}, NCD_{sub}) and MULLET (T_{1trans}, T_{2trans}) (Cheng and DeGiorgio, 2019), obtained by averaging the scores with various window sizes and a step size of 10 nucleotides. The data were generated with SLiM on the tree shown in Figure 4 (C) under BS.
